# The neurodegenerative effects of selenium are inhibited by FOXO and PINK1/PTEN regulation of insulin/insulin-like growth factor signaling in *Caenorhabditis elegans*^?^

**DOI:** 10.1016/j.neuro.2013.12.012

**Published:** 2014-03

**Authors:** Annette O. Estevez, Kathleen L. Morgan, Nathaniel J. Szewczyk, David Gems, Miguel Estevez

**Affiliations:** a Department of Neurology, University of Arizona College of Medicine, Tucson, AZ 85724, USA; b Veterans Affairs Pittsburgh Healthcare System, Research and Development (151U), University Drive C, Pittsburgh, PA 15240, USA; c Department of Biological Sciences, University of Pittsburgh, Pittsburgh, PA 15260, USA; d Institute of Healthy Ageing, and Department of Genetics, Evolution, and Environment, University College London, The Darwin Building, Gower Street, London WC1E 6BT, UK

**Keywords:** Selenium, Neurodegeneration, Insulin/insulin-like signaling, PTEN, PINK1, Amyotrophic lateral sclerosis

## Abstract

Exposures to high levels of environmental selenium have been associated with motor neuron disease in both animals and humans and high levels of selenite have been identified in the cerebrospinal fluid of patients with amyotrophic lateral sclerosis (ALS). We have shown previously that exposures to high levels of sodium selenite in the environment of *Caenorhabditis elegans* adult animals can induce neurodegeneration and cell loss resulting in motor deficits and death and that this is at least partially caused by a reduction in cholinergic signaling across the neuromuscular junction. Here we provide evidence that reduction in insulin/insulin-like (IIS) signaling alters response to high dose levels of environmental selenium which in turn can regulate the IIS pathway. Most specifically we show that nuclear localization and thus activation of the DAF-16/forkhead box transcription factor occurs in response to selenium exposure although this was not observed in motor neurons of the ventral cord. Yet, tissue specific expression and generalized overexpression of DAF-16 can partially rescue the neurodegenerative and behavioral deficits observed with high dose selenium exposures in not only the cholinergic, but also the GABAergic motor neurons. In addition, two modifiers of IIS signaling, PTEN (phosphatase and tensin homolog, deleted on chromosome 10) and PINK1 (PTEN-induced putative kinase 1) are required for the cellular antioxidant reduced glutathione to mitigate the selenium-induced movement deficits. Studies have suggested that environmental exposures can lead to ALS or other neurological diseases and this model of selenium-induced neurodegeneration developed in a genetically tractable organism provides a tool for examining the combined roles of genetics and environment in the neuro-pathologic disease process.

## Highlights

• Insulin/insulin-like signaling reduction alters selenium-induced neurodegeneration.
• Selenium induces nuclear translocation of DAF-16/FOXO3a.
• DAF-16 overexpression decreases GABAergic and cholinergic motor neuron degeneration.
• Loss of DAF-18/PTEN increases sensitivity to selenium-induced movement deficits.
• Glutathione requires DAF-18/PINK-1 to improve selenium-induced movement deficits.

## 1 Introduction

Selenium (Se) is an essential micronutrient with a narrow recommended dietary range. Insufficient dietary Se can be lethal contributing to the cardiomyopathy of Keshan Disease and the osteochondropathy of Kashin-Beck disease (Lee and Jeong, 2012). And, while supranutritional levels of Se can also be lethal (Spiller and Pfiefer, 2007), high levels of selenite [Se(IV)] and other Se compounds such as Ebselen have been used effectively as cancer therapeutics (Sanmartín et al., 2012; Fairweather-Tait et al., 2011). During the course of a number of cancer treatment trials, it was observed that the higher doses of Se capable of reducing the incidence of various cancers were positively associated with increases in fasting glucose levels and type 2 diabetes (Rocourt and Cheng, 2013; Steinbrenner, 2013). Based on these and other studies, potential mechanisms have been proposed for how Se levels alter diabetic status and include a role for selenoproteins (Rayman and Stranges, 2013; Labunskyy et al., 2011). Selenoproteins are proteins that incorporate Se in the form of selenocysteine and include the antioxidant proteins: glutathione peroxidase (GPx), thioredoxin reductase (TrxR), and selenoprotein P (SeP). Increased levels of SeP have been observed in diabetic and pre-diabetic humans (Yang et al., 2011), and in animal models overexpression of GPx induced insulin resistance (McClung et al., 2004). Targeted knockouts of these selenoproteins in animals models are associated with neurodegeneration (Caito et al., 2011; Wirth et al., 2010), and lower levels are found in individuals with Alzheimer's and other neurological diseases (Vinceti et al., 2013; Zhang et al., 2010).

Supranutritional levels of Se can also exert effects on the insulin/insulin-like growth factor (IGF)-1 signaling (IIS) pathway via the induction of hydrogen peroxide (H_2_O_2_) and reactive oxygen species (ROS) formation (Steinbrenner, 2013). High levels of exogenously applied H_2_O_2_ prevent phosphorylation/activation of AKT1/PKB (Iwakami et al., 2011), a serine/threonine kinase that is a downstream mediator of IIS signaling (Alessi et al., 1996). Similarly, levels of Se which induce ROS formation prevent AKT phosphorylation (Luo et al., 2012, 2013; Pinto et al., 2011; Wu et al., 2006) or induce its dephosphorylation (Wu et al., 2006). By preventing AKT activation, the downstream forkhead box O transcription factors (FOXO) are not phosphorylated which leads to their activation and subsequent nuclear translocation. Increased incidences of metabolic disorders, including diabetes are associated with transcriptional activation of FOXO proteins (Barthel et al., 2005). In addition, FOXO proteins are known to regulate expression of pro-apoptotic and oxidative stress response genes (Eijkelenboom and Burgering, 2013) which inhibit cancers and aging (Huang and Tindall, 2007) and their activation has also been shown to be neuroprotective (Mojsilovic-Petrovic et al., 2009) and lead to the transcription of SeP (Walter et al., 2008). A mutation in the FOXA2 gene was also significantly associated with the sporadic form of the motor neuron disease, amyotrophic lateral sclerosis (ALS) (Dunckley et al., 2007).

Environmental exposures to high levels of Se, have led to the development of a neurological disorder in both humans and animals that bears striking similarity to ALS (Vinceti et al., 2010; Panter et al., 1996). More recently, Vinceti et al. (2013) reported significantly higher levels of Se(IV) in the cerebrospinal fluid of neurologically impaired patients that were later confirmed to have ALS. Additionally, they reported that SeP levels were reduced in these patients. This coupled with the identification of the FOXA2 association with sporadic ALS, as mentioned above implies that FOXO transcriptional regulation may be altered in ALS. In fact, the *Caenorhabditis elegans* FOXO3a ortholog, DAF-16 was shown to be required for lifespan extension and improved movement by reducing the toxic effects of TDP-43 (transactive response DNA binding protein 43) and SOD1 (superoxide dismutase 1) (Boccitto et al., 2012; Vaccaro et al., 2012; Zhang et al., 2011) which have both been linked to sporadic and familial forms of ALS (Sreedharan et al., 2008; Kabashi et al., 2008; Rosen et al., 1993; Jones et al., 1993). The importance of FOXO in other neurodegenerative diseases has been demonstrated in both *C. elegans* and elsewhere (Wong et al., 2013; Parker et al., 2012; Maiese et al., 2007; Morley et al., 2002).

The free-living soil nematode *C. elegans* has one selenoprotein, a TrxR ortholog TRXR-1 (Gladyshev et al., 1999) which is not required for Se-induced lethality (Boehler et al., 2013). Since selenoproteins do not play a direct role in Se toxicity within *C. elegans*, it is an ideal model to examine how Se alters other cellular processes within a eukaryotic organism. The nervous system of *C. elegans* hermaphrodites consists of 302 neurons and 56 support cells, and contains all of the classic neurotransmitters observed in higher organisms including serotonin, dopamine, acetylcholine, ?-aminobutyric acid (GABA), and glutamate (Altun and Hall, 2011). Thus, *C. elegans* is an excellent model to study neurodegenerative disease (Dimitriadi and Hart, 2010). We have shown previously that in adult animals high dose exposures to Se in the form of sodium selenite [Se(IV)] induce reactive oxygen species (ROS) formation and motor neuron degeneration in *C. elegans* which results in alterations in the normal movement behaviors leading to paralysis and death (Estevez et al., 2012; Morgan et al., 2010). These phenotypes could be partially ameliorated by exogenous exposure to the cellular antioxidant reduced glutathione (GSH) (Estevez et al., 2012; Morgan et al., 2010). Animals with mutations in genes altering both cholinergic and GABAergic signaling were more sensitive to Se(IV) [i.e. the mutations were shown to further reduce normal movement in Se(IV) exposed animals], and reduced cholinergic signaling across the neuromuscular junction (NMJ) was determined to be partly responsible for these phenotypic changes which mimics aspects of ALS pathology (Estevez et al., 2012). Here we show that reductions in IIS signaling which lead to activation of DAF-16/FOXO3a are neuroprotective against these Se(IV)-induced effects. In addition we show evidence that Se(IV) causes degeneration and loss of GABAergic motor neurons, and that the loss of both the cholinergic and GABAergic neurons with high dose Se(IV) exposure was partially dependent on the levels of DAF-16 expression within the animals. Here we also report that animals with mutations in genes encoding for PTEN (phosphatase and tensin homolog, deleted on chromosome 10) and PINK1 (PTEN-induced putative kinase 1) are resistant to the positive improvements on the Se(IV)-induced movement behaviors previously observed with exogenous application of GSH. This GSH resistance phenotype was only previously seen in animals with a mutation in the dithiol glutaredoxin gene *glrx-21* (Estevez et al., 2012; Morgan et al., 2010). The model of Se(IV) toxicity presented here can be used to further elucidate the link between increased Se status and alterations in IIS signaling to disease processes affecting insulin resistance and type 2 diabetes, and cancer, as well as neurodegenerative disorders such as ALS, Parkinson's and Alzheimer's diseases.

## 2 Materials and methods

### 2.1 Strains, maintenance, and growth conditions

The following strains were used: **N2**: *wild-type*(*WT*) variety, Bristol, **CB1370**: *daf-2(e1370)III*, **CB1375**: *daf-18(e1375)IV*, **CB5600**: *ccIs4251 [pSAK2 (Pmyo-3::GFP-LacZ(NLS))* + *pSAK4 (Pmyo-3::mitochondrial GFP)* + *dpy-20(+)]I; him-8(e1489) IV*, **CF1038**: *daf-16(mu86)I*, **CF1442**: *daf-16(mu86)I;daf-2(e1370)III; muEx169 [*pNL206*(Punc-119::gfp::daf-16a)* + pRF4[*rol-6(su1006)*]*]*, **CF1514**: *daf-16(mu86)I;daf-2(e1370)III; muEx211 [*pNL213*(Pges-1::gfp::daf-16a)* + pRF4 [*rol-6(su1006)*]*]*, **CF1515**: *daf-16(mu86)I;daf-2(e1370)III; muEx212 [*pNL212*(Pmyo-3::gfp::daf-16a)* + pRF4[*rol-6(su1006)*]*]*, **DR26**: *daf-16(m26)I*, **DR1564**: *daf-2(m41)III*, **GR1310**: *akt-1(mg144)V*, **OM148**: *daf-2(e1370)III; yzIs71[Ptph-1::gfp* + pRF4[*rol-6(su1006)*]*]V*, **OM249**: *daf-16(mu86)I; daf-2(e1370)III; yzIs71[Ptph-1::gfp* + pRF4[*rol-6(su1006)*]*]V*, **OM261**: *vsIs33[dop-3::rfp]V; wuIs56[Psod-3::sod-3::gfp* + pRF4[*rol-6(su1006)*]*]*, **OM285:** *daf-16(mu86)I; yzIs71[Ptph-1::gfp* + pRF4[*rol-6(su1006)*]*]*, **OM324:** *zIs356[*pGP30*(Pdaf-16::daf-16a2::gfp)* + pRF4[*rol-6(su1006)*]*]IV; vsIs33[Pdop-3::rfp]V*, **OM325** *daf-16(mu86)I; vsIs33[Pdop-3::rfp]V; wdIs20[snb-1::GFP]*, **RB712**: *daf-18(ok480)IV*, **RB2547**: *pink-1(ok3538)II*, **TJ356**: *zIs356[*pGP30*(Pdaf-16::daf-16a2::gfp)* + pRF4[*rol-6(su1006)*]*]IV*, **TJ1052**: *age-1(hx546)II*. The *daf-16(mu86)I;daf-2(e1370)III* animals examined in Fig. 3A were obtained by selecting non-roller adult animals from those strains expressing the daf-16-tissue specific extra-chromosomal arrays. Animals that did not roll were no longer expressing the extra-chromosomal array and were used as a control for determining the effects of the double mutation on the Se(IV)-induced motility deficits.

The following caveat must be noted in regards to the *sod-3::GFP* transgene used here. Expression of *sod-3* accounts for only 1% of the total *sod* mRNA levels in *C. elegans* adults, thus the strong expression exhibited by the *sod-3::gfp* transgene examined here is most likely artifactual, but as reported is very likely to express the native pattern (Doonan et al., 2008). Several laboratories have reported similar bright expression patterns with the generation of other P*sod-3* transgenes (Wolf et al., 2008; Libina et al., 2003).

All strains were maintained and grown at 20 °C on modified NGM plates without additional calcium as described (Estevez et al., 2004) and all experiments were performed at 20 °C unless otherwise stated.

### 2.2 Movement assay

Adult animals were developmentally synchronized and placed on plates containing 5 mM sodium selenite (Spectrum Chemicals, Gardena, CA) alone or with 3 mM glutathione (Sigma–Aldrich, St. Louis, MO) as described (Estevez et al., 2012; Morgan et al., 2010). Mock-exposed animals (controls) were placed on plates containing an equivalent amount of carrier (dH_2_O) to that used on the experimental plates. After plating, animals were scored at 24-h intervals for the movement behaviors as previously described (Morgan et al., 2010). For experiments performed at 25 °C, developmentally synchronized animals at the fourth larval stage (L4) were placed on plates overnight in order to develop to the adult stage at 25 °C. They were transferred the next day to fresh plates with or without the addition of Se(IV) as described (Morgan et al., 2010). The percentage motile (%motile) indicates the number of animals moving normally after a tap to the head and tail as previously described (Morgan et al., 2010).

### 2.3 Heat-shock

Developmentally synchronized adult animals expressing the *Pdaf-16::gfp* construct, *zIs356* (Henderson and Johnson, 2001) were placed on plates with 5 mM Se(IV) or an equivalent volume of dH_2_O (mock-exposed control) for 2 h at 20 °C then shifted to 34 °C for 30 min (Lin et al., 2001) before being fluorescently imaged; or they were directly visualized following the 2 h incubation.

### 2.4 Fluorescence imaging

Following either mock or Se(IV) exposure, ten live animals at a time were anesthetized by placing them into a drop of M9/tricaine solution (0.01% tricaine/0.001% tetramisole) on a glass microscope slide. A coverslip was placed over the animals prior to visualization. Three to five slides (30–50 animals) were visualized for each experiment. Because the animals were visualized alive, only those animals that remained alive and intact were documented.

Animals were visualized on an Olympus BX51 microscope equipped with epifluorescence (Olympus America Inc., Center Valley, PA) and utilizing a FITC (GFP) or Texas Red (RFP) filter. Images were documented with a QImaging Retiga 1300 camera and imaging system (QImaging, Surrey, British Columbia, Canada). Processing of images for publication was accomplished by the Adobe CS5 Software package (Adobe Systems Inc., San Jose, CA) utilizing both Photoshop and Illustrator to create the final images.

For the studies examining the effects of the proteasomal inhibitors levamisole and the MG-132 (Z-Leu-Leu-Leu-CHO), CB5600 animals were simultaneously exposed to Se(IV) and either of the drugs for 48 h. The concentrations of the drugs were as described in Szewczyk et al. (2000).

### 2.5 Statistical analysis

Statistical analysis was performed by one-way ANOVA with post hoc analysis by the Bonferroni–Holm method using Microsoft Excel 2010 (Microsoft Inc., Seattle, WA) with the add-in Daniel's XL Toolbox version 5.6 (http://xltoolbox.sourceforge.net). Student's *t*-test was two-tailed with unequal variance.

## 3 Results

### 3.1 Mutations in the insulin/insulin-like growth factor pathway alter sensitivity/resistance to environmental selenium

**Fig. 1:**
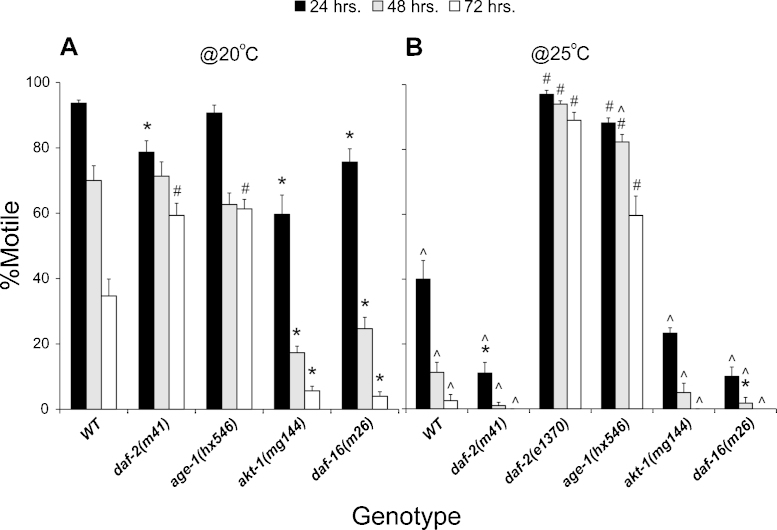
Mutations in the IGF pathway affect selenium sensitivity. Animals containing mutations in the *daf-2* IGF pathway show an altered sensitivity to Se(IV)-induced motility effects that is enhanced by increasing temperature. (A) When grown at 20 °C, adult animals were found to be more resistant (#) or to exhibit significantly increased sensitivity (*) to Se(IV) in comparison to the WT strain by one-way ANOVA performed across each time point (*p* = 0.05; significance was determined by post hoc analysis using the Bonferroni–Holm method). (B) The phenotypes (sensitivity or resistance) observed at 20 °C were significantly enhanced by Se(IV)-exposure at 25 °C at some or all of the time points tested for all strains [^ significantly different at 25 ° when each strain was compared to the same strain and time exposure at 20 °C; *p* < 0.05 by Student's *t*-test (two tailed, unequal variance)]. Comparisons of the mutant strains to WT when all were grown at 25 °C, showed significant increases in resistance (#) or sensitivity (*) in the animals at some or all of the time points tested. Analysis was by one-way ANOVA performed across each time point (*p* = 1.4 × 10^-11^; significance was determined by post hoc analysis using the Bonferroni–Holm method). When not exposed to Se(IV), all strains had =95% motility at each time point and temperature tested (data not shown), with the only exception being *akt-1* which exhibited 80% motility across all time points at 25 °C only (data not shown). Each bar graph represents the average of three populations (*n* = 150 at 20 °C; *n* = 60 at 25 °C) with error bars indicating SEM. WT = N2 strain; Se = Se(IV). All mutations are reduction-of-function mutations with the exception of *mg144* which is an activating mutation in the *akt-1* gene.

Populations of wild-type (WT) adult *C. elegans* animals exposed to sodium selenite [Se(IV)] in their growth media exhibit a decline in the percentage of normal moving animals that is both dose and time dependent (Morgan et al., 2010). An exposure of 5 mM Se(IV) was shown to lead to a progressive decline in movement from mild impairment (backing deficient) to paralysis, and death, and to induce oxidative stress and cholinergic motor neuron degeneration (Estevez et al., 2012; Morgan et al., 2010), all of which mimics aspects of amyotrophic lateral sclerosis (ALS). ALS is a neurodegenerative disease characterized by motor neuron loss in the brain and spinal cord and which typically results in death within five years from diagnosis. The causes of ALS are multifaceted and include both genetic factors, and environmental influences (Trojsi et al., 2013). High dose Se exposures have been implicated in ALS-like disease in both animals and humans (Vinceti et al., 2010). Because alterations in insulin/insulin-like growth factor signaling (IIS) have also been associated with ALS, we wanted to determine if alterations in IIS signaling could affect the response of adult animals in our high dose Se(IV) assay. To first determine if alterations in the levels of IIS signaling components can affect the movement behavior of *C. elegans* adults, animals containing mutations in genes encoding components of the IIS signaling pathway were exposed over time to 5 mM Se(IV) and their movement accessed. When grown at optimal growth temperature (20 °C – Fig. 1A), animals with a reduction-of-function (*rf*) mutation, *m41* in the *daf-2* gene which encodes for the *C. elegans* insulin/insulin-like growth factor receptor (IGFR) (Kimura et al., 1997), were more resistant to the Se(IV)-induced reduction in normal motility at 72 h in comparison to WT (#, *m41*, Fig. 1A), although at 24 h they were initially more sensitive (*, *m41*, Fig. 1A). Both *hx546* a *rf* mutation in the *age-1* gene [which encodes for the phosphoinositide 3-kinase (PI(3)K) (Morris et al., 1996) downstream of DAF-2 (Ogg et al., 1997)] and the *rf* mutation *sa709* in the worm PDK1 encoding gene *pdk-1*, conferred increased resistance to the Se(IV)-induced motility impairment at 72 h (#, *hx546*, Fig. 1A; data not shown for *pdk-1*, *p* = 2.2 × 10^-3^ by one-way ANOVA comparing *sa709* to WT at 72 h). In contrast, an activating mutation in the gene encoding for the *C. elegans* ortholog of the serine/threonine kinase AKT/PKB, *akt-1(mg144)* (Paradis and Ruvkun, 1998) and an *rf* mutation in the forkhead box transcription factor (FOXO3a) gene, *daf-16(m26)* (Ogg et al., 1997; Lin et al., 1997) significantly increased the sensitivity of these animals to Se(IV) by reducing the number of animals moving normally in comparison to the WT strain (*, *mg144* and *m26*, Fig. 1A). The increased sensitivity to Se(IV) observed with these two strains was manifest across all the time points tested. Thus, reductions in IIS signaling are protective against the decline in normal movement behavior previously observed to occur when WT adult animals were exposed to high levels of environmental Se(IV).

### 3.2 The effects of high dose selenium exposure on movement is enhanced with increased temperature

Stressors including oxidative stress and heat can reduce IIS signaling which leads to activation of DAF-16/FOXO3a (Landis and Murphy, 2010; Honda and Honda, 2002). We have shown that high dose Se(IV) exposures also result in increased stress as indicated by the detection of reactive oxygen species (ROS) formation in exposed animals and that increased temperature reduced movement in WT animals (Morgan et al., 2010). To determine whether the alterations in normal movement observed within populations of the IIS mutant animals exposed to high dose Se(IV) could be enhanced by increased temperature (i.e. increased stress), animals grown at 20 °C until the adult stage were exposed to Se(IV) at 25 °C. Many of the original components of the IIS signaling pathway were isolated as temperature sensitive (*ts*) regulators of a developmental process that can lead to the formation of an alternate third stage larva known as a dauer larva (Golden and Riddle, 1984; Riddle et al., 1981; Swanson and Riddle, 1981; Riddle, 1977), and 25 °C is a temperature at which the *ts* dauer larva formation phenotype is fully penetrant in many of the IIS mutant strains (Swanson and Riddle, 1981). At this higher temperature, the WT animals, those with the *rf* mutations *daf-2(m41)* or *daf-16(m26)*, or the *akt-1(mg144)* activating mutation had significantly fewer normal moving animals at all of the time points when compared to animals of the same genotype exposed at 20 °C for the given 24 h time period (^, *p* < 0.05: Fig. 1B). Thus, there was an overall enhancement of the sensitivity to the Se(IV)-induced effects on movement when these animals were exposed at the higher temperature. Similarly, the populations of *age-1(hx546)* animals were observed to have a significantly greater number of normal moving animals within their populations, but only when they were exposed for 48 h at 25 °C (^, *p* < 0.05: Fig. 1B). This was an enhancement of the resistance phenotype observed at 48 h for the *age-1(hx546)* animals exposed at 20 °C. Finally, when compared to the WT strain exposed to Se(IV) at 25 °C, all of the IIS mutant strains exposed under the same conditions exhibited the same Se(IV) responsive phenotype that they exhibited at 20 °C [i.e. more sensitive or resistant to Se(IV) in comparison to WT] with the exception of *akt-1(mg144)* which was no different than WT at this temperature (*mg144*, Fig. 1B). Thus, we show that the additional stressor of increasing temperature during Se(IV)-exposure results in enhancement of the Se(IV)-induced movement phenotypes that were observed with exposures at 20 °C, and that high dose Se(IV)-exposure induces a stress response process that appears to be mediated by IIS signaling similar to its control of other stress response pathways (Shore and Ruvkun, 2013).

### 3.3 *daf-2* alleles have opposite selenium responsive phenotypes under high temperature stress

There are over 40 *daf-2* alleles that have been isolated and these have been described to express a range of phenotypes in response to environmental and oxidative stressors (Ackerman and Gems, 2012; Honda and Honda, 2002; Scott et al., 2002; Gems et al., 1998). Most recently, Vaccaro et al. (2012) showed that the *e1370* allele enhanced neurodegeneration in a worm TDP43/TDP-1 ALS model while another allele *e1368* suppressed the neurodegeneration. The *m41* allele has been previously described as thermolabile or a true temperature-sensitive allele (Gems et al., 1998) and has demonstrated differences in phenotype from the hypomorphic alleles of *daf-2* in regards to life span, intrinsic thermal tolerance, and enhancements of its Daf-c phenotype by mutations in the tyrosine phosphatase-like *sdf-9* (Gems et al., 1998; Jensen et al., 2007). Here we observed that *m41* allele of *daf-2* was more sensitive to the Se(IV)-induced effects on normal movement when exposed at 25 °C, a phenotype it shared with the *daf-16(m26)* animals (Fig. 1B). Yet, at 20 °C although it was also initially more sensitive, after 72 h of exposure time the *daf-2(m41)* animals were more resistant to Se(IV)’s effect on normal movement; a phenotype opposite to that of the *daf-16* mutant animals exposed under the same conditions (Fig. 1A). Because of this discrepancy in phenotypes exhibited by the *m41* allele after Se(IV)-exposure, we tested an additional allele *e1370* for its effect on movement behavior during the combined Se(IV) and heat-induced stress conditions. The *e1370* allele was previously shown to be more resistant to the combined effects of oxidative stress and hyperoxia, a phenotype that was opposite to and suppressed by a mutation in the *daf-16* gene (Honda and Honda, 2002). Here we similarly observed that the *daf-2(e1370)* animals were significantly and consistently more resistant to the Se(IV)-induced reductions in normal movement when compared to WT animals grown under the same conditions at 25 °C (#, *e1370*, Fig. 1B). Because the combined stressors of high heat and high dose Se(IV) exposure enhanced the phenotypes of the other IIS signaling component mutants, it is very likely that the phenotype expressed by the *daf-2(e1370)* allele under these conditions is more indicative of the phenotype that other *daf-2* alleles (other than *m41*) would be expected to express.

### 3.4 Selenium induces nuclear translocation of DAF-16/FOXO3a

**Fig. 2:**
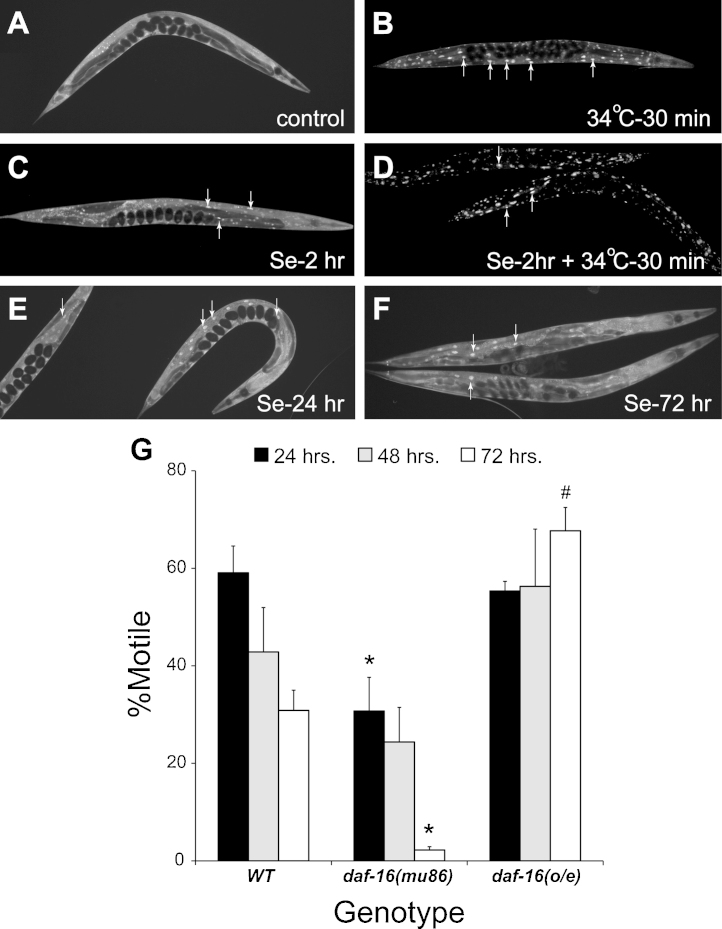
DAF-16 nuclear localization and dosage effects on motility with selenium exposure. Se(IV) induces DAF-16::GFP to translocate to the nucleus which is required for protection from the Se(IV)-induced effects on motility. (A–F) A strain (TJ356) containing a transgene (*zIs356*) that overexpresses wild-type DAF-16::GFP under control of the *daf-16* promoter (Henderson and Johnson, 2001) was examined to determine its subcellular localization under various conditions. (A) Well-fed animals maintained at 20 °C show cytoplasmic expression of DAF-16::GFP. (B) Heat-shock-induced stress results in nuclear localization of DAF-16 (indicated by arrows) as previously described (Henderson and Johnson, 2001; Lin et al., 2001). (C) Se(IV) exposure induced DAF-16::GFP translocation after 2 h. (D) Exposure to Se(IV) for 2 h followed by heat shock increased nuclear translocation. (E and F) Longer exposures to Se(IV) (without heat shock) did not vastly increase localized GFP expression. All animals were maintained in a well-fed state at 20 °C and were exposed in the presence of food. Magnification = 100×. (G) In comparison to the WT strain, the presumptive null-mutation, *daf-16(mu86)* conferred significantly more sensitivity to the Se(IV)-induced effects on motility at 24 and 72 h [*, sensitivity to Se(IV), *p* = 8.0 × 10^-3^] while animals overexpressing DAF-16 [*daf-16(o*/*e)* = *zIs356*] were significantly more resistant at 72 h [# resistance to Se(IV); *p* = 4.3 × 10^-6^] by one-way ANOVA performed across each time point with significance determined by post hoc analysis using the Bonferroni–Holm method. Each graph bar represents at least three populations of 20 animals per strain (*n* = 60); error bars indicate SEM. Se = Se(IV).

DAF-16 has been observed to translocate into the nucleus in response to starvation, heat shock, and oxidative stressors (Henderson and Johnson, 2001; Lin et al., 2001). Here we have shown that reduction in IIS signaling coupled with high dose Se(IV) exposure reduces the percentage of normal moving animals and that this can be enhanced when exposure occurs at an increased temperature. Since reduction in IIS signaling can activate DAF-16 and reduction of *daf-16* function leads to increased Se(IV)-sensitivity (Fig. 1), we hypothesized that high dose Se(IV) exposure would lead to nuclear translocation of DAF-16. In order to investigate this, we examined a strain TJ356 that overexpresses the DAF-16a2 isoform linked to the green fluorescent protein (GFP) and which is driven by the *daf-16* promoter (Henderson and Johnson, 2001). Under normal growth conditions (well-fed and grown at 20 °C) expression of this DAF-16::GFP reporter is diffuse and primarily localized to the cytoplasm (Fig. 2A), as was reported previously (Henderson and Johnson, 2001). Translocation of the DAF-16::GFP to the nucleus occurs with mild heat shock (34 °C for 30 min) (Henderson and Johnson, 2001; Lin et al., 2001). It was observed here that mild heat shock was capable of inducing nuclear translocation in adults as evidenced by the localized punctate expression of the GFP although some cytoplasmic localization was still observed (Fig. 2B). Our previous studies had shown that exposures to high dose Se(IV) could induce phenotypic changes in the normal movement and egg-laying behaviors of adult animals in as early as 6 h (Estevez et al., 2012; Morgan et al., 2010). Here we observed translocation of DAF-16::GFP to the nucleus within only 2 h after exposure to Se(IV) although cytoplasmic localization was still apparent (Fig. 2C). Henderson and Johnson (2001) similarly showed that the localization of DAF-16::GFP after exposure to the pro-oxidant juglone was not as strong as when animals were exposed to heat shock. Since we had shown that in WT animals coupling Se(IV) exposure with increased temperature could enhance the Se(IV)-induced reduction in normal movement (Fig. 1B; Morgan et al., 2010), we expected that exposing animals to Se(IV) for 2 h followed by mild heat shock would enhance the nuclear localization of DAF-16. As expected, when the 2 h Se(IV) exposure was followed by heat shock, the majority of the DAF-16 expression was observed to be located in the nucleus (Fig. 2D). This nuclear localization was stronger than when animals were exposed to mild heat shock (Fig. 2B) or Se(IV) alone (Fig. 2C). Thus, as predicted exposure to high dose Se(IV) levels lead to activation of DAF-16 similar to that previously observed to occur with reductions in IIS signaling. This activation could be enhanced by the additional stress of high temperature exposure as was observed for the movement behaviors.

### 3.5 DAF-16 is required to alleviate the selenium induced effects on normal movement

WT adult animals chronically exposed to high dose Se(IV) levels experienced behavioral deficits that increased with the time of exposure (Estevez et al., 2012; Morgan et al., 2010). Similarly, it was shown here that the percentage of normal moving animals continued to decrease over time when Se(IV)-exposure was coupled with IIS signaling reduction (Fig. 1A). Thus, it was expected that increasing the time of exposure to high dose Se(IV) would also increase the number of cells with activated DAF-16 that was localized to the nucleus. This was not the case. Instead increasing the exposure time to 24 (Fig. 2E) or 72 h (Fig. 2F) did not alter the expression pattern from that observed after the 2 h Se(IV) only exposure (Fig. 2C). One possible explanation for this unexpected result is that with continued exposure to Se(IV), a greater percentage of animals die or are significantly impaired (Morgan et al., 2010), thus only animals that are experiencing less Se(IV)-induced stress are able to survive the photo-documentation process. To circumvent this issue we examined the effect of high dose Se(IV)-exposure over time on animals experiencing different levels of DAF-16 expression and examined their motility (Fig. 2G). *daf-16(mu86)* is a deletion allele that is a presumptive null mutation (Lin et al., 1997). Animals with this mutation were significantly more sensitive to the effects of Se(IV) on movement than the animals with WT levels of DAF-16 at both 24 h and 72 h after Se(IV) exposure (*, Fig. 2G; Table 1). This was similar to the levels observed with the *daf-16 rf* allele, *m26* (Fig. 1A). Thus, loss of DAF-16 leads to a predicted sensitivity to high dose Se(IV). In contrast, overexpression of DAF-16 [*daf-16(o/e)*] improves motility over time since significantly more animals are moving normally within this population than in populations of animals with WT levels of DAF-16 at the 72 h exposure time point (#, Fig. 2G). Thus, increasing the expression level of DAF-16 is capable of minimizing the Se(IV)-induced declines in normal movement observed over time with Se(IV)-exposure.

### 3.6 Tissue-specific expression of DAF-16 partially rescues the daf-16-deficient selenium sensitivity

DAF-16 is natively expressed in all tissue types except some pharyngeal cells (Henderson and Johnson, 2001). The prior experiment demonstrated that overexpression of DAF-16 in native tissue is capable of partially alleviating the Se(IV)-induced effects on motility (Fig. 2G), but could not address which specific tissue, if any DAF-16 was exerting this effect. Previous studies showed that expression of DAF-16 in intestines could partially restore the reduced lifespan of *daf-16(-)* animals (Libina et al., 2003).

**Fig. 3:**
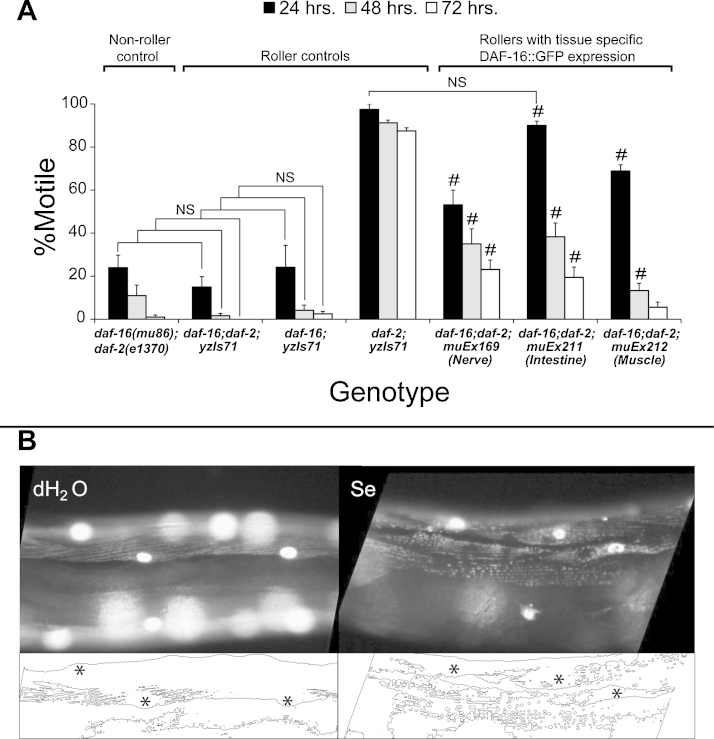
Selenium exposure and the effects of tissue-specific expression of DAF-16 on motility. Overexpression of DAF-16 in nerves, intestines, or muscles of adult animals resulted in an increased resistance to the Se(IV)-induced motility defects. (A) Animals expressing the DAF-16 tissue-specific constructs were more resistant (#) to the Se(IV)-induced effects on motility when compared to animals with the same genetic background (*daf-16;daf-2*) and that expressed the same reporter plasmid, pRF4. This plasmid contained within the *yzIs71* transgene induces animals to roll in place (roller phenotype) rather than move forward or backward in a normal sinusoidal wave pattern, a phenotype that results from the expression of a dominant mutation (*su1006*) in the *rol-6* gene (Mello et al., 1991). No significant difference (NS) was observed between the *daf-16;daf-2* roller and non-roller strains at all temperatures, between the *daf-16* and the *daf-16;daf-2* roller strains at all temperatures, nor the *daf-2* roller strain and the *daf-16;daf-2* intestine expressing strain at 24 h only (as determined by one-way ANOVA). Significance is defined as *p* = 0.01 as determined by one-way ANOVA followed by post hoc analysis using the Bonferroni–Holm method. All non-Se(IV)-treated strains had a greater than 90% survival at all times tested (data not shown). Each bar graph represents the average of six populations of 20 animals per strain (*n* = 120); error bars ± SEM. *daf-16* = *daf-16(mu86)*; *daf-2* = *daf-2(e1370)*; *daf-16*;*daf-2*;*yzIs71* = *OM249*; *daf-16*;*yzIs71* = *OM285*; *daf-2*;*yzIs71* = *OM148*. (B) Upper panel: Animals of the strain CB5600 transiently express mitochondrial GFP in their muscles and are shown here after 48 h of either mock- (dH_2_O) or Se(IV)-(Se)exposure. The GFP pattern is tubular indicating healthy mitochondria in the water control (dH_2_O). Se(IV)-exposure results in fragmentation of the GFP expression indicating damage to the muscle mitochondria (Se). Lower panel: Black and white outline of GFP expression patterns above, nuclei are represented by asterisks for orientation. Se = Se(IV).

To determine whether expression of DAF-16 in specific tissues was capable of improving the movement of animals exposed to high dose Se(IV), we took advantage of the availability of strains CF1442, CF1514, and CF1515 (Table 1 and Section 2.1) which express GFP::DAF-16 under control of promoters for the nervous system (*muEx169*), intestines (*muEx211*), and muscles (*muEx212*), respectively (Libina et al., 2003). All of these strains were constructed to have the same genetic background and include the mutations *daf-16(mu86)* and *daf-2(e1370)*. The presence of the *mu86* mutation presumably eliminates any native DAF-16 expression since it is believed to be a null mutation in the *daf-16* gene (discussed in Section 3.5). Thus, DAF-16 expression in each of these strains would be limited to the specific tissues controlled by the promoter regulating its expression. In addition, each of the transgenes also contain a reporter plasmid pRF4 expressing the mutation *su1006*, an allele of a collagen gene *rol-6* which results in a dominant rolling (Rol) phenotype in the animals that express this mutation. Rolling movement is different than normal sinusoidal movement therefore we had to confirm that the Rol phenotype had no effect on the Se(IV)-induced movement deficits. To accomplish this we generated a strain (OM249; Section 2.1) that expresses a different transgene (*yzIs71*), but contains the same reporter plasmid pRF4, and is in the same genetic background *daf-16(mu86);daf-2(e1370)* as the strains expressing the tissue specific transgenes (Roller control: *daf-16;daf-2;yzIs71*; Fig. 3A). When compared to *daf-16(mu86);daf-2(e1370)* animals containing no transgene (Non-roller control; Fig. 3A), the OM249 animals (Roller control: *daf-16;daf-2;yzIs71*; Fig. 3A) were not significantly different (NS; Fig. 3A) over all the time points tested. In addition, there was also no difference between OM249 and OM285 (Section 2.1) a strain that was identical to OM249, but lacking the *daf-2* mutation (Roller control: *daf-16;yzIs71*; Fig. 3A). These data suggest that the Rol phenotype did not affect the Se(IV)-induced movement deficits.

Next animals from each of the three tissue specific DAF-16 expression strains (CF1442, CF1514, CF1515; Rollers with tissue specific DAF-16::GFP expression: Fig. 3A; Table 1) were exposed to Se(IV) and compared at all the time points to both OM249 (Roller control: *daf-16;daf-2;yzIs71*; Fig. 3A) and the *daf-16;daf-2* animals expressing no transgene (Non-roller control; Fig. 3A). All of the strains expressing the tissue specific transgenes showed significant improvement when compared to OM249 (#, Fig. 3A) as well as to the *daf-16;daf-2* non-roller control animals (Table 1), but with a few exceptions (NS, *p* value against non-transgene, Table 1). The most notable improvement was observed at the 24 h exposure time point in animals expressing DAF-16 in the intestines (CF1514: Table 1). When the CF1514 animals were compared to either the *daf-2* non-roller (CB1370, Table 1) or *daf-2* roller animals (Roller control: *daf-2;yzIs71*; Fig. 3A), the CF1514 animals were not significantly different (NS; Table 1 and Fig. 3A). Both of these *daf-2* strains exhibit the Se(IV)-resistance phenotype previously observed for the *daf-2(e1370)* allele (Fig. 1B) and do not have a mutation in the *daf-16* gene, thus they express WT levels of DAF-16. This demonstrates that the Se(IV)-induced movement deficits observed in animals lacking DAF-16 could be fully suppressed at 24 h by intestinal expression of DAF-16 in a *daf-16;daf-2* mutant background. After 24 h, intestinal suppression was still significantly improved, but the percentage of normal moving animals within the population trended downward (Fig. 3A). This trend downward was observed over time for all three of the DAF-16 tissue specific strains when exposed to Se(IV) and with the exception of the 72 h muscle specific expression, they all still maintained a significant improvement over the *daf-16;daf-2* non-roller and roller control strains, (Fig. 3A and Table 1). Thus, expression of DAF-16 in the nerves, intestines, or muscles is capable of at least partially preventing the Se(IV)-induced effects on movement with the greatest improvement observed in the intestines at 24 h.

### 3.7 Selenium cause mitochondrial damage to muscles

Muscle damage after exposure to high levels of Se is well documented in livestock and humans (Davis et al., 2012; Spiller and Pfiefer, 2007; Tiwary et al., 2006), but in *C. elegans* the body wall and egg-laying muscles were shown to be functionally and structurally intact after 48 h of exposure to high dose Se(IV) (Estevez et al., 2012). Yet, here we have demonstrated that muscle specific expression of DAF-16 can lead to partial rescue of normal movement (Fig. 3A) suggesting that Se(IV) does affect muscle in *C. elegans*. Recent studies have shown that activation of FOXO3a leads to ROS accumulation in the mitochondrial membrane which is followed by additional accumulation of FOXO3a in the nucleus and later more intense mitochondrial ROS accumulation culminating in apoptosis (Hagenbuchner and Ausserlechner, 2013). If a similar cascade of events is occurring in the muscle after Se(IV) exposure than it is possible that the reduction in normal movement observed after the 48 h time point in animals with DAF-16 muscle specific expression may be due to the accumulation of mitochondrial damage. To examine this possibility, we used a strain CB5600 which expresses GFP in the mitochondria and nuclei of muscle and exposed the animals for 48 h to Se(IV) (Fig. 3B). Since after the 48 h time point Se(IV)-exposed animals experienced increased lethality and fragility, it was not possible to examine these older animals. At 48 h, the mitochondria of the water treated age-matched controls exhibited normal tubular shaped expression (H_2_O, Fig. 3B) while the GFP expression of the mitochondria in Se(IV) exposed animals was observed to be patchy and disorganized an indication that fragmentation of the mitochondrial network had occurred (Se, Fig. 3B). Although levamisole and MG-132 (Z-Leu-Leu-Leu-CHO) have been shown to block proteasome mediated degradation in muscle (Muñoz-Lobato et al., 2013; Estevez et al., 2012), they were not able to here block the Se(IV)-induced mitochondrial fragmentation (Supp. Fig. 1). These data demonstrate that Se(IV) exposure is capable of inducing mitochondrial damage to muscle and that this damage is not prevented by proteasomal blockade.

### 3.8 DAF-16 prevents motor neuron degeneration

**Fig. 4:**
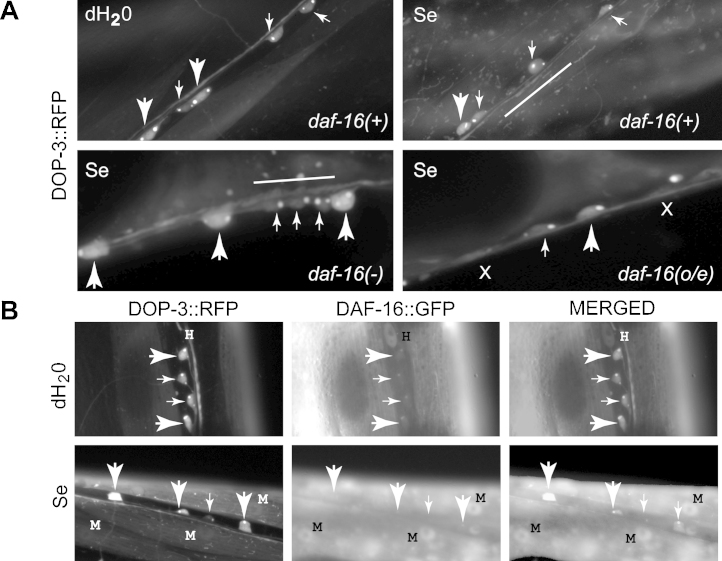
Selenium exposure and the dosage effects of DAF-16 on neurodegeneration. The level of DAF-16 expression affects the observed levels of neurodegeneration in the ventral cord. (A) The DOP-3::RFP construct (*vsIs33*) expresses in both cholinergic (small arrows) and GABAergic (large arrows) motor neurons, but more weakly expresses in the former than the latter (Chase et al., 2004). In animals with WT levels of DAF-16 [*daf16(+)* = strain OM261], the normal fusiform shape of the motor neuron cell bodies observed in the control animals [*daf-16(+)*, dH_2_O] is altered in animals exposed to Se(IV) [*daf-16(+)*, Se]. Axonal blebbing of the ventral cord (line) is also observed with Se(IV) exposure, but not in the control animals; as was previously reported (Estevez et al., 2012). With loss of DAF-16 [*daf-16(-)* = strain OM325], neuronal damage is increased. Over-expression of DAF-16 [*daf-16(o*/*e)* = strain OM324] reduced the blebbing in the cord as well as cell body rounding. Cell loss was still observed in the DAF-16 over-expressing animals. This had been shown previously to occur in WT animals exposed to Se(IV) (Estevez et al., 2012). (B) Se(IV) does not induce DAF-16::GFP (*zIs356*) localization to the nucleus in either the cholinergic (small arrows) or GABAergic motor neurons (large arrows) of adult animals (strain = OM324), but was observed in muscle cells (M). Nuclear localization of DAF-16 in the hypodermal cells (H) of animals not exposed to Se(IV) (dH_2_O) is most likely due to stress induced on the animals which were alive, but paralyzed while being imaged, as previously observed (Lin et al., 2001). Magnification = 400×. Se = Se(IV). The genotype of each strain is listed in Section 2. Animals shown here are representative of 30–50 animals examined under.

Because expression of DAF-16 in neurons partially rescued the Se(IV)-sensitivity phenotype of *daf-16(mu86)*, we examined whether this partial rescue was due to activation of DAF-16 in the motor neurons specifically. In animals expressing *vsIs33*, cholinergic motor neurons weakly express RFP making them easily identifiable from the GABAergic motor neurons which strongly express RFP under control of the promoter for the *dop-3* gene which encodes for the *C. elegans* ortholog of the mammalian D2 dopamine receptor (Chase et al., 2004). In animals expressing *dop-3::rfp* in a WT *daf-16* background [*daf-16(+)*; Fig. 4A], Se(IV)-exposure induced the cholinergic motor neurons (small arrows, Fig. 4) to round and lose their fusiform shape, and blebbing of the ventral cord (white lines, Fig. 4A), as had been previously described (Estevez et al., 2012). Reduction in GABAergic signaling through the UNC-49 receptor was shown to decrease the percentage of forward moving animals after Se(IV)-exposure (Estevez et al., 2012) and here we show that the GABAergic motor neurons (large arrows, Fig. 4) showed signs of neurodegeneration similar to that observed in the cholinergic motor neurons (small arrows, Fig. 4). Loss of DAF-16 [*daf-16(-)*; Fig. 4A] seemed to enhance these Se(IV) induced effects. The over-expression of DAF-16 [*daf-16(o*/*e)*; Fig. 4A] under control of the *daf-16* promoter was partially protective against these changes since axonal blebbing was reduced, as was cell-rounding although evidence of cell loss was still apparent (**x**, Fig. 4A). Cytoplasmic and nuclear localization of DAF-16::GFP has been reported in the neurons of *daf-16(+)* animals grown under standard conditions (Henderson and Johnson, 2001) and specifically in the ventral cord neurons of dauer larvae (Lin et al., 2001). In the animals overexpressing DAF-16, the protein did not appear to be localized to the nucleus in the motor neuron cells (compare dH_2_O and Se, Fig. 4B), but was expressed in the nuclei of muscle cells (M, Fig. 4B) after Se(IV) exposure. Together, these data demonstrate that although Se(IV) does not cause detectable nuclear translocation of the DAF-16::GFP within motor neurons, some of the neurotoxic effects induced by Se(IV) on motor neurons can be ameliorated by increased global expression of DAF-16.

### 3.9 Mutation in the DAF-16 target gene *sod-3* leads to selenium-resistance

**Fig. 5:**
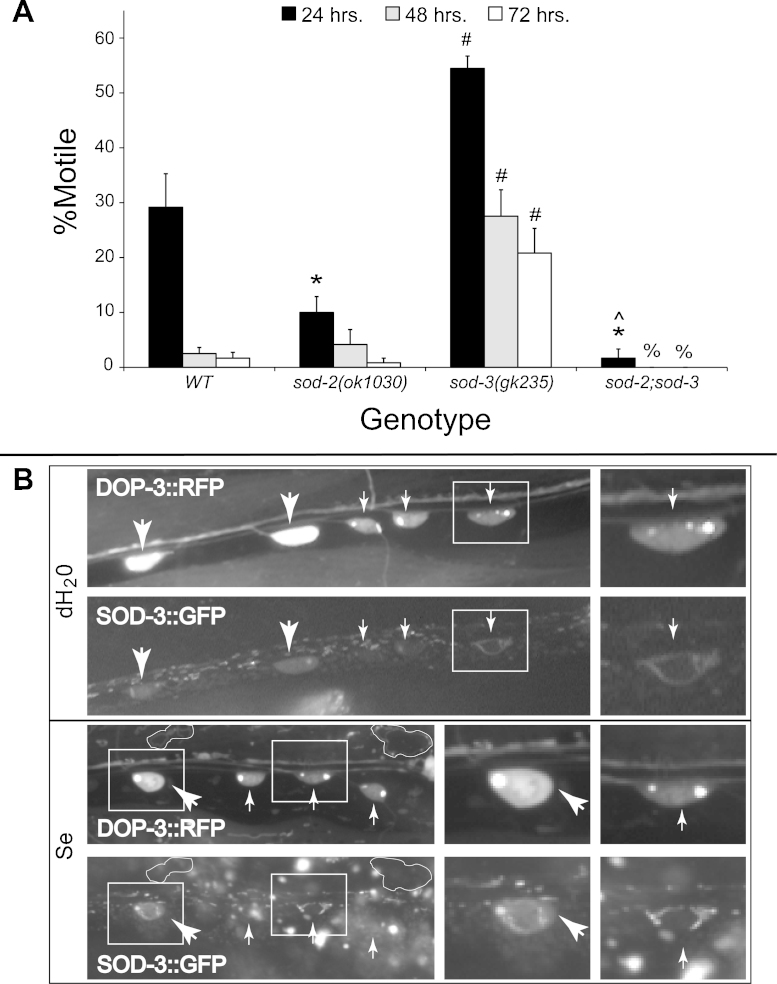
The effects of mutations on downstream target genes of DAF-16 transcription. *C. elegans* encodes for two superoxide dismutase 2 (SOD2) genes which are regulated by DAF-16 and have opposite effects on the Se(IV)-induced movement deficits. (A) Animals with a mutation in the *sod-2* gene are initially more sensitive to the effects of Se(IV) than WT animals (at 24 h: *p* = 3.8 × 10^-3^ by one-way ANOVA), but at later time points are no different than WT animals (*p* = 0.5) while animals with a *sod-3* mutation are consistently and significantly more resistant to Se(IV)’s effect on movement than WT (*p* = 0.03 by one-way ANOVA across all time points tested). The *sod-2;sod-3* double mutant animals are phenotypically similar to the *sod-2* single mutant animals, the animals are initially more sensitive than WT to Se(IV) at 24 h (*p* = 4.3 × 10^-4^ by one-way ANOVA), but are no different at the later time points (*p* > 0.1). Animals were grown at 25 °C, but the trends and significances were the same for animals grown at 20 °C (data not shown). (B) Animals of the strain OM261 which expresses DOP-3::RFP and over-expresses a SOD-3::GFP translational fusion under control of its native promoter (*wuIs56*) were exposed to either water (dH_2_O) or Se(IV) (Se) for 24 h. The SOD-3::GFP was observed to co-localize with DOP-3::RFP to the ventral cord and the motor neurons (large arrows = GABAergic; small = cholinergic), but was unable to protect either from the Se(IV)-induced neurodegenerative effects. This SOD-3::GFP expression is discontinuous in the Se(IV)-exposed motor neurons (Se, SOD-3::GFP, right panels) in comparison to the water exposed ones (H_2_O, SOD-3::GFP, right panel) suggesting that mitochondrial fragmentation has occurred as was observed in the Se(IV)-exposed muscle (Fig. 3B). In the Se(IV) exposed animals, muscles are observed to exhibit increased expression of DOP-3::RFP that did not colocalize with the SOD-3::GFP expression (Se, white outline) and was not observed in the water controls. Smaller panels on the right focus in on the motor neurons that are boxed in the larger image immediately to their left. Magnification = 400×. Se = Se(IV).

The superoxide dismutases (SOD) are required for oxidative stress responses in many organisms including *C. elegans* (Honda et al., 2010; Doonan et al., 2008). There are three general types of SOD: copper/zinc (Cu/Zn SOD or SOD1), manganese (Mn-SOD or SOD2), and extracellular (EC-SOD or SOD3) (Miao and St Clair, 2009). Deletion of SOD2 in mice leads to progressive motor weakness and neurodegeneration (Lebovitz et al., 1996). In addition, SOD2 has been implicated in mitochondrial dysfunction and examined for its role in models of neurodegenerative diseases including stroke, ALS, and Parkinson's, Alzheimer's, and Huntington's Diseases (Flynn and Melov, 2013; Maier and Chan, 2002; Andreassen et al., 2000). In *C. elegans*, there are two Mn-SODs encoded by the *sod-2* and *sod-3* genes (Hunter et al., 1997) which have been shown to be transcriptionally regulated by DAF-16 (Cabreiro et al., 2011; Oh et al., 2006). Knockout/reduction mutations in the *sod-3* gene typically have no effect or are sensitivity to oxidative stressors such as paraquat and juglone (Back et al., 2012), but have been shown to be more resistant to cadmium toxicity (Roh et al., 2009); similar results have been reported for *sod-2* mutations in regards to stress (Back et al., 2012). When exposed to high dose Se(IV), animals with a mutation in *sod-2* were more sensitive after 24 h of exposure in comparison to WT animals (*p* = 3.8 × 10^-3^, Fig. 5A, *) while those with a mutation in *sod-3* exhibited resistance across all time points tested (*p* = 0.02; Fig. 5A, #) with regards to their movement phenotype. The Se(IV)-resistance exhibited by the *sod-3* mutant animals was suppressed by the *sod-2* mutation in *sod-2;sod-3* double mutant animals, and the double mutant strain was more sensitive to Se(IV) than was *sod-2* alone (*p* = 4.2 × 10^-4^, compared at the 24 h time point; Fig. 5A). Increased sensitivity for *sod-2;sod-3* double mutant animals was previously reported for stress induced by paraquat-hyperoxia and juglone treatments (Back et al., 2012). Thus, loss of *sod-3* or *sod-2* had opposite phenotypes in regards to their response to high dose Se(IV)-exposure while the *sod-2;sod-3* double mutant exhibited a phenotype similar to the *sod-2* mutation alone.

Since animals with loss of SOD-3 expression are resistant to the movement deficits normally elicited by high dose Se(IV) exposure, we hypothesized that SOD-3 overexpression would not protect motor neurons from Se-induced neurodegeneration. To determine this we examined the ventral cord of animals overexpressing SOD-3 translationally fused to GFP (Fig. 5B). These animals coexpressed the *dop-3::rfp* transgene examined previously (Fig. 4) allowing for ease in identifying the GABAergic (large arrows, Fig. 5B) and cholinergic (small arrows, Fig. 5B) motor neurons. SOD-3::GFP expression was observed in both types of motor neurons and along the ventral cord in the mock-exposed animals (dH_2_O, Fig. 5B). After 24 h of exposure to Se(IV), the SOD-3::GFP expression pattern appeared fragmented within the motor neurons (Se, Fig. 5B, larger images and smaller panels to the right). Since SOD-3 was shown to co-localize with mitochondrial staining (Wolf et al., 2008), this fragmentation pattern most likely reflects mitochondrial fragmentation similar to that observed in the muscle (Fig. 3B). In addition, signs of degeneration including rounding of the motor neurons and blebbing of the ventral cord were observed with Se(IV) exposure (Se, Fig. 5B). Thus, as predicted by the loss-of-function *sod-3* phenotype, SOD-3 overexpression did not prevent the neurodegeneration previously observed to occur in the GABAergic (Fig. 4) and cholinergic (Fig. 4; Estevez et al., 2012) motor neurons after high dose Se(IV)-exposure.

### 3.10 Selenium exposure increases expression of the DOP-3/D2 dopamine receptor in muscle

Muscles expressing the DOP-3::RFP and exposed to Se(IV), showed increased expression levels of DOP-3::RFP not observed in the muscles of water exposed animals (Fig. 5B; compare DOP-3::RFP, dH_2_O to Se). This expression did not appear to co-localize with the SOD-3::GFP expression [Fig. 5B, outlined regions in Se(IV)-exposed animals]. Expression of DOP-3::RFP in muscles has been previously reported as weak (Chase et al., 2004). No previous studies to our knowledge have described DOP-3 expression patterns in muscles under stress. Here we demonstrate that stress induced by high dose Se(IV) exposure increased expression of DOP-3 in the muscle.

### 3.11 Deletion of daf-18/PTEN leads to selenium sensitivity

**Fig. 6:**
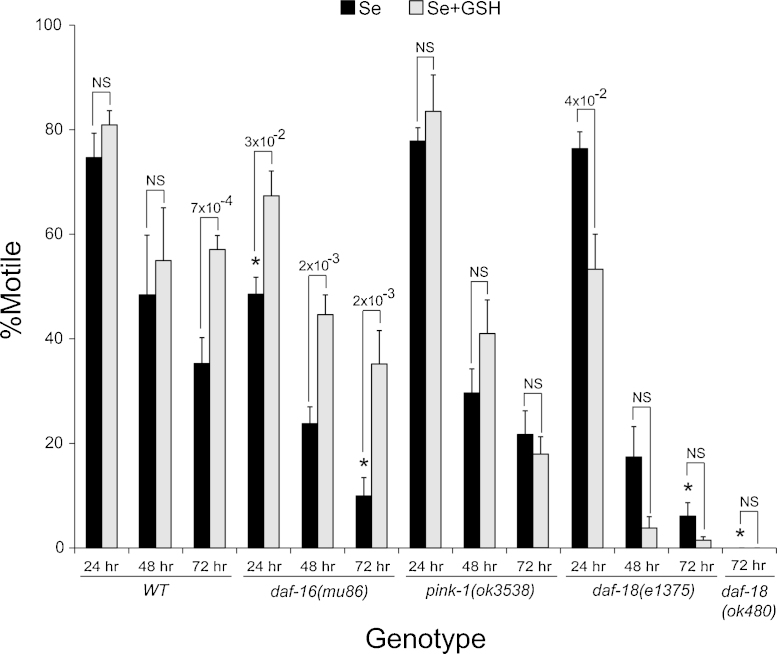
The cellular antioxidant glutathione and its effect on modulators and a downstream regulator of IGF signaling when exposed to selenium. Animals containing mutations in the genes encoding the *C. elegans* orthologs of PTEN (phosphatase and tensin homolog, *daf-18*) and PINK1 (PTEN-induced putative kinase, *pink-1*), modulators of the IGFR signaling are not rescued from the Se(IV)-induced motility defects by exposure to the cellular antioxidant glutathione (GSH), but the Se(IV)-sensitivity observed with the *daf-16(mu86)* mutation was rescued. All the strains were sensitive to Se(IV), but increased sensitivity to Se(IV) (*, *p* = 0.05) was not observed until 24 h of exposure in the animals with a mutation in *daf-16*, and at 72 h in the *daf-16* and *daf-18(e1375* and *ok480*) mutant animals when compared to *wild-type* (*WT*). Analysis was by one-way ANOVA performed across each time point [significance (indicated above bar graphs) was determined by post hoc analysis using the Bonferroni–Holm method; NS = not significant]. Glutathione was able to significantly rescue the Se(IV)-induced deficits in WT animals after 72 h of continuous exposure (significance is indicated above each pair of bars and was determined by Student's *t*-test). When not exposed to Se(IV), all strains had =95% motility at each time point and temperature tested (data not shown). The addition of GSH [without Se(IV)] did not alter motility in comparison to the control (water only exposed) animals (data not shown). Each bar graph represents the average of three populations (*n* = 60) with error bars indicating SEM. Se = Se(IV).

Mutations in PTEN (phosphatase and tensin homolog deleted on chromosome 10) and PINK1 (PTEN-induced putative kinase 1) have been linked to neurodegenerative disease processes affecting ALS, Parkinson's and Alzheimer's Diseases (Domanskyi et al., 2011; Kirby et al., 2011; Wilhelmus et al., 2011; Morimoto et al., 2010; Sonoda et al., 2010; Valente et al., 2004). PTEN has been shown to regulate neuronal outgrowth and induces apoptotic cell death in neurons (Wong et al., 2013; Christensen et al., 2011) by inhibiting PI(3)K-dependent activation of AKT (Stambolic et al., 1998) allowing for the activation of FOXO transcription. Conversely, PINK1 is required for the IIS dependent activation of AKT (Akundi et al., 2012) which leads to the phosphorylation inactivation of FOXO. Because of their opposite roles in regulating FOXO transcription, we hypothesized that mutations in the *C. elegans* orthologs of *PTEN* and *PINK1*, *daf-18* and *pink-1*, respectively, would have opposite phenotypes in regards to their responsiveness to high dose Se(IV) exposure: *daf-18*/*PTEN* mutants would be expected to express a sensitivity phenotype similar to that observed with loss or reduction of *daf-16* while PINK1 mutants should be more resistant to Se(IV). As expected, animals with the *daf-18* mutations *e1375(rf)* and *ok480(lf)* were both more sensitive although this sensitivity was not observed until 72 h after exposure to high dose Se(IV) (*, Fig. 6). This sensitivity phenotype was similar to previous studies in which it was demonstrated that genetic or RNA inhibition of *daf-18* resulted in increased sensitivity to the oxidative stressor paraquat (Masse et al., 2008; Honda and Honda, 2002). Animals with a mutation in the *pink-1(ok3538)* were no more sensitive or resistant to Se(IV) than was WT (Fig. 6). Thus, although PINK-1 does not appear to be required for Se(IV) to induce its neurotoxic effects on movement in *C. elegans*, loss of *daf-18* does increase sensitivity to Se(IV) suggesting that it plays a protective role against Se(IV)-induced toxicity.

### 3.12 Glutathione requires DAF-18 and PINK-1 to ameliorate the selenium-induced movement deficits

The cellular antioxidant reduced glutathione (GSH) plays an essential role in protecting against the damaging effects ROS. In *C. elegans*, exogenous GSH has been shown to reduce oxidative stress and to partially rescue the movement and egg-laying deficits caused by Se(IV)-exposure in WT animals (Estevez et al., 2012; Morgan et al., 2010). Previously, we showed the dithiol glutaredoxin, GLRX-21 was required for GSH to exert these protective effects (Estevez et al., 2012; Morgan et al., 2010). When *daf-16(mu86)* animals were concomitantly exposed to Se(IV) and GSH, they responded no differently than WT, i.e. GSH was able to partially rescue the movement deficits observed with Se(IV) exposure alone at all the time points tested (Fig. 6) as was previously shown for WT animals (Morgan et al., 2010). This improvement was observed across all the time points tested for the *daf-16 lf* animals. Although the improvement observed in WT animals was only significant at the 72 h time point here, we had previously observed differences at both 24 and 48 h depending on dosage (Morgan et al., 2010). Thus, this data suggests that DAF-16 is not required for GSH to provide protection from high dose Se(IV) exposure.

Hydrogen peroxide induced oxidation of PTEN has been shown to be reduced by glutathione in yeast and human cells (Kim et al., 2011) while oxidative stress significantly increased levels of GSH in cells from patients with a missense mutation in PINK1 in comparison to controls (Grünewald et al., 2009). When animals with the *pink-1* or *daf-18* mutations were treated with GSH, they did not respond favorably; none of these animals showed improved percentages of animals moving normally after treatment (Fig. 6). In fact when both sets of animals were exposed to Se(IV), at 24 h the GSH-treated *daf-18(e1375)* animals showed a significant reduction in the percentage of animals moving normally in comparison to animals not treated with GSH (Fig. 6). At later time points, both *daf-18* mutant animals showed a trend downward in response to GSH treatment although it was not significant (Fig. 6). Thus, both PTEN and PINK1 appear to be required for GSH to ameliorate the Se(IV)-induced motility deficits.

## 4 Discussion

**Fig. 7:**
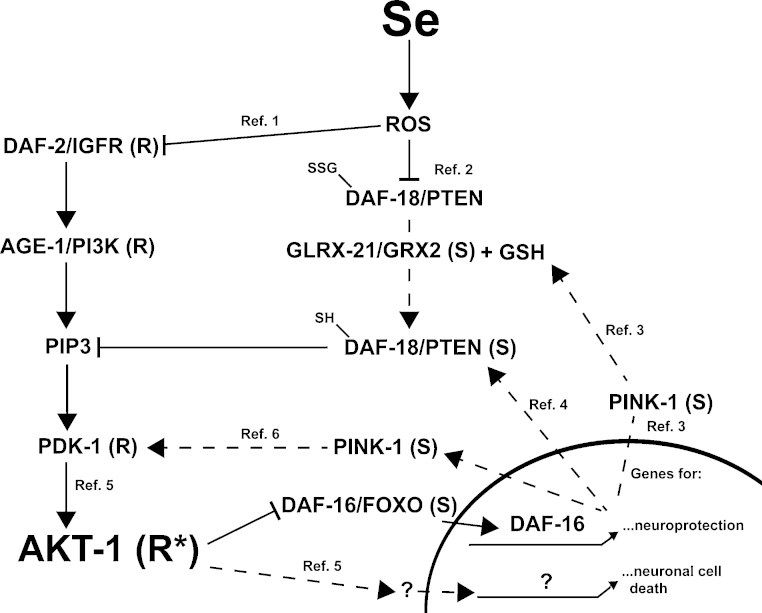
Summary model of the effects of sodium selenite in *C. elegans*. Adult animals with mutations in components of the DAF-2/IGFR signaling cascade responded to high dose Se(IV) within their environment similar to that observed when exposed to other stressors (Honda and Honda, 2002), i.e. the loss- or reduction-of-function *daf-16* alleles had an opposite phenotype from that of many of the upstream components that inhibit its nuclear translocation. Yet, although the pattern was predictable, the specific sensitivity or resistance phenotypes were not. A model depicting the effects of Se(IV) on the IGFR signaling cascade including modulatory branches is shown and summarizes the data presented here as well as previously by us (Morgan et al., 2010), and elsewhere (Luo et al., 2013; Akundi et al., 2012; Ponugoti et al., 2012; Kim et al., 2011; Loh et al., 2009; Mei et al., 2009; Budovskaya et al., 2008). The Se(IV) (selenite) responsive phenotypes, listed in parentheses as R (resistant) or S (sensitive), are based on the phenotypes of the reduction-of-function mutants. *R, resistance phenotype of reduction-of-function mutant is assumed since the *mg144* gain-of-function mutation is sensitive (Fig. 1); Dotted lines represent pathways that are assumed based on data observed in other organisms, but not confirmed in *C. elegans*. IGFR = insulin/insulin growth factor-like receptor; PI3K = phosphatidylinositide 3-kinase; FOXO = forkhead box transcription factor; PTEN = phosphatase and tensin homolog; GSH = reduced glutathione; GSSG = oxidized glutathione; Ref. 1 = Ponugoti et al. (2012); Ref. 2 = Kim et al. (2011), Loh et al. (2009); Ref. 3 = Mei et al. (2009); Ref. 4 = Luo et al. (2013); Ref. 5 = Budovskaya et al. (2008); Ref. 6 = Akundi et al. (2012).

We have shown previously that exposures to high levels of selenium [Se(IV)] in the environment of adult animals can induce neurodegeneration and cell loss resulting in motor deficits and death (Estevez et al., 2012; Morgan et al., 2010) and that this is at least partially caused by a reduction in cholinergic signaling across the neuromuscular junction (Estevez et al., 2012). Here we provide evidence that a reduction in the insulin/insulin-like (IIS) stress response pathway regulates the response to high dose levels of environmental selenium which in turn regulates the IIS pathway. Most specifically we show that nuclear localization and thus activation of the DAF-16/forkhead box transcription factor occurs in response to Se(IV) exposure although this was not observed in motor neurons of the ventral cord. Yet, tissue specific expression and generalized overexpression of DAF-16 can partially rescue the neurodegenerative and behavioral deficits observed with high dose Se(IV) exposures. In addition, we determined that two modifiers of AKT activation, PINK1 and PTEN are required for the cellular antioxidant glutathione to mitigate the Se(IV)-induced movement deficits, previously shown to also require the dithiol glutaredoxin, GLRX-21 (Morgan et al., 2010). The data presented here and previously are summarized in Fig. 7.

Overexpression of DAF-16 under native promoter control leads to the partial rescue of the Se(IV)-induced effects on movement and neurodegeneration (Table 1 and Fig. 2G; Fig. 4A) while loss of *daf-16* had the opposite effect on both phenotypes (Table 1; Fig. 2G). This suggests a neuroprotective role for DAF-16 during Se(IV)-induced toxicosis in *C. elegans* (Fig. 7). Gene expression through activation of DAF-16 prevents ROS-induced neuronal degeneration (Calixto et al., 2012), as well as protects from neurodegeneration in worm models of Huntington's (Parker et al., 2012), and motor neuron diseases (Mojsilovic-Petrovic et al., 2009). Yet, surprisingly neuron-specific expression of DAF-16 did not fully rescue the movement deficits (Fig. 3A). Limiting expression of DAF-16 to the intestines on the other hand fully rescued the movement deficits, but only for the first 24 h after exposure (Fig. 3A). If DAF-16 can function non-cell autonomously as previously suggested (Iser et al., 2007; Libina et al., 2003), then it is possible that intestinal expression of DAF-16 could result in secretion of a diffusible factor that activates DAF-16 within the motor neurons preventing the movement deficits normally observed with Se(IV) exposure (Morgan et al., 2010). Yet, the inability to detect DAF-16 nuclear localization in motor neurons (Fig. 4B) after Se(IV) exposure although localization in other cell types was observed (Fig. 2C–F; Fig. 4B) does not seem to support this notion, but suggests another possibility. Activation of DAF-16 in hypodermal tissue was observed by Qi et al. (2012) to affect germ-line proliferation without activating DAF-16 in the gonad, thus DAF-16 activation in the intestine may similarly non-autonomously protect the motor neurons without activating DAF-16 in these neurons. Our observation that the level of DAF-16 expression could affect the amount of damage to the motor neurons and the ventral cord with Se(IV)-exposure (Fig. 4A) without inducing detectable translocation of DAF-16 into the neurons (Fig. 4B) seems to support this hypothesis.

The *sod-3* gene is reported to be a direct target of the DAF-16 transcriptional activation (Oh et al., 2006) and is required by DAF-16 for oxidative stress response (Honda et al., 2010). Yet, if activation of the *sod-3* gene occurred in response to DAF-16 transcription, than *sod-3* mutant animals would be expected to have the same phenotype as the *daf-16(-)* animals in response to Se(IV)-exposure. We did not observe this to be the case since the *sod-3* mutant animals were resistant to Se(IV) while the *daf-16* mutant animals were more sensitive (compare Fig. 5A to Figs. 1A and 2G). In addition, overexpression of SOD-3 did not appear to prevent the Se(IV)-induced neurodegeneration in the motor neurons (Fig. 5B) although overexpression of DAF-16 did partially rescue this effect (Fig. 4A). Given this result it seems unlikely that *sod-3* expression is activated by DAF-16 in response to high dose Se(IV) exposures. In addition, transcriptional repression of *sod-3* expression by DAF-16 is unlikely since SOD-3 expression was observed in both the GABAergic and cholinergic motor neurons (Fig. 5B) and DAF-16 was not (Fig. 4B). Thus, the data presented here would suggest that SOD-3 expression in the motor neurons is regulated by a transcription factor other than DAF-16. Indeed SOD-3 expression in other cells has been reported to be regulated by the GATA transcription factor ELT-3 (Budovskaya et al., 2008). Further studies aimed at examining motor neuron expression of SOD-3::GFP in the background of animals with mutations in other transcription factors would address this issue, but is outside the scope of this paper.

PINK1 and PTEN have both been shown to be induced by FOXO3a transcription (Luo et al., 2013; Mei et al., 2009) and although it is possible that such an interaction occurs in worms, it has not been demonstrated. Here we report that loss or reduction of either *daf-18/PTEN* or *pink-1* results in a decreased percentage of normal moving animals when either strain is exposed to Se(IV). Yet although the *daf-18/PTEN* animals were significantly more sensitive to Se(IV) than was WT [i.e. *daf-18* mutant animals were Se(IV)-sensitive], the *pink-1* animals were no worse than WT (Fig. 6). The similarity of the phenotypes displayed by the *daf-18/PTEN* and the *daf-16* mutant animals in response to Se(IV) exposure suggests the possibility that *daf-18* is transcriptionally regulated by *daf-16* (Fig. 7) although further studies would need to be performed to confirm this. PINK1 was initially identified as a PTEN inducible kinase (Valente et al., 2004) which was more recently shown to be regulated by PTEN through its inhibition of AKT signaling and subsequent activation of FOXO3a transcription of the *PINK1* gene (Mei et al., 2009). In addition to being induced by PTEN, PINK1 has also been shown to induce AKT phosphorylation and the subsequent inactivation of FOXO3a (Fig. 7) (Akundi et al., 2012). Thus, loss of PINK1 in *C. elegans* may not induce an increased Se(IV)-sensitivity phenotype simply because phosphorylation of AKT by PINK1 can be achieved by other kinases including PDK-1 and mTOR (Sánchez-Mora et al., 2012; Murata et al., 2011; Maj et al., 2010; Paradis et al., 1999).

The loss of *pink-1* or *daf-18*/*PTEN* was here shown to produce a GSH insensitivity phenotype (Fig. 6) which had only previously been observed by loss of GLRX-21, the glutaredoxin 2 (GRX)2-like protein (Estevez et al., 2012; Morgan et al., 2010). ROS has been demonstrated to oxidize and thus inactivate PTEN and this process requires both GRX and GSH to be reduced (Kim et al., 2011). Thus, a similar mechanism may occur in *C. elegans* accounting for the GSH insensitivity phenotype of the *daf-18* mutants (Fig. 7). Loss of PINK1 was observed to lead to a dramatic decrease in GSH levels (Mei et al., 2009), as well as an increase in the activity of the enzyme glutathione S-transferase (GST) (Hoepken et al., 2007). GST has been shown to increase in response to high levels of sodium selenite (Sun et al., 2013; Zhang et al., 2008). Thus, the inability of exogenous GSH to improve movement in the *pink-1* mutant animals may reflect an increase in catabolism of GSH through the increased activities of GST. Further studies examining the GST levels in both WT and *pink-1* mutants would be able to address this issue in more depth.

Selenite can induce mitochondrial fragmentation in glioma cells (Kim et al., 2007) and was shown here to induce mitochondrial fragmentation in muscles and neurons (Figs. 3B and 5B, respectively). Overexpression of Mn-SOD was shown to prevent this selenite-induced mitochondrial damage in the glioma cells, but in *C. elegans* overexpression of the SOD-3/Mn-SOD protein did not (Fig. 5B). The increased expression of the D2-like dopamine receptor DOP-3 observed in muscle after Se(IV) exposure (Fig. 5B) might represent an attempt to alter the mitochondrial damage induced by Se(IV) even though the DOP-3::RFP was not observed to co-localize with the SOD-3 expression in the mitochondria (compare outlines Fig. 5B). Examining the effects of D2 receptor activation in a Parkinson's model, Chau et al. (2009) showed receptor activation improved mitochondrial membrane potential and up-regulated intracellular levels of GSH. Thus the upregulation of DOP-3 observed in the muscle may be an attempt to stabilize the mitochondria and prevent mitochondrial damage. This question could not be directly addressed within the context of these studies since the DOP-3 construct used was a promoter fusion only and not a rescuing clone (Chase et al., 2004).

## 5 Conclusion

Here we present more extensive evidence that high dose exposures to sodium selenite can induce neurodegeneration and demonstrate damage of both the cholinergic and GABAergic motor neurons in the ventral cord. Reductions in both cholinergic and GABAergic signaling were previously shown to alter normal movement. Similarly, movement deficits were observed to occur with reductions in insulin/insulin-like signaling that are known to activate DAF-16/forkhead box-3a and increased expression of DAF-16 was shown to both improve movement and reduce neurodegeneration. This suggests that DAF-16 alters expression of genes that are neuroprotective against the toxic insult of high dose selenium exposure. Two gene products that are known to be regulated by forkhead box transcription, PINK1 and PTEN were shown here to be required for the cellular anti-oxidant reduced glutathione to reduce the neurodegenerative effects on movement while overexpression of a third gene, *sod-3* known to be regulated in *C. elegans* by DAF-16 did not alter the neurodegenerative process. Taken together these data suggest a link between toxicant exposures and complex regulation of signaling processes that can alter the course of neurological decline. That environmental exposures can lead to ALS or other neurological diseases is suspected through previous observations, and this model of selenium-induced neurodegeneration developed in a genetically tractable organism provides a tool for examining the combined roles of genetics and environment in neuro-pathologic disease progression.

## Conflict of interest statement

None declared.

## Figures and Tables

**Supplemental Fig. I:**
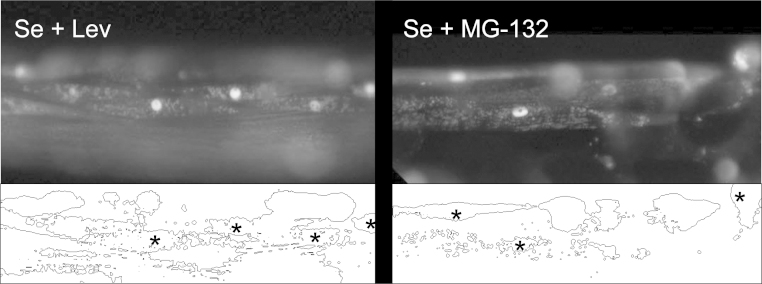
Inhibition of proteasome does not prevent Se(IV)-induced damage to mitochondria. Upper panel: Animals of the strain CB5600 transiently express mitochondrial GFP in their muscles that became fragmented after Se(IV) exposure for 48 h (Fig. 1). This damage could not be prevented by inhibition of proteasomal degradation with either levamisole (Se + Lev) or MG-132 (Se + MG-132) when adult animals were exposed simultaneously to Se(IV) and either compound for 48 h. Lower panel: Black and white outline of GFP expression patterns above, nuclei are represented by asterisks for orientation. Lev = 1 mM levamisol; MG-132 = 200 µM Z-Leu-Leu-Leu-CHO (MG-132). Se = Se(IV).

**Table 1:** Motility of selenium exposed animals with tissue-specific *daf-16* expression.

Strain name	Genetic background	Transgene	Phenotype	Time (h)	Mean motility ± SEM (%)	*p* value^a^ against non-transgene^b^	*p* value^a^ against *daf-2*	*p* value^a^ against *daf-16*
N2	*WT*	None	Non-roller	24	65.5 ± 5.5	–	–	–
				48	42.8.0 ± 6.5	–	–	–
				72	32.0.0 ± 3.5	–	–	–
CB1370	*daf-2 (e1370)*	None		24	97.0 ± 1.2	–	–	–
				48	94.0 ± 1.0	–	–	–
				72	89.0 ± 2.4	–	–	–
CF1038	*daf-16 (mu86)*	None		24	39.6 ± 5.2	–	–	–
				48	23.8 ± 3.2	–	–	–
				72	6.1 ± 1.9	–	–	–
None^c^	*daf-16;daf-2*	None		24	24.0 ± 5.8	–	1.7 × 10^-6^	NS
				48	11.0 ± 4.8	–	1.6 × 10^-7^	NS
				72	1.0 ± 1.0	–	7.3 × 10^-10^	NS
TJ356	*WT*	*zIs356* (native)	Roller	24	55.3 ± 2.0	NS	–	–
				48	56.3 ± 11.8	NS	–	–
				72	67.7 ± 4.8	1.5 × 10^-6^	–	–
CF1442	*daf-16;daf-2*	*muEx169* (nerve)		24	53.1 ± 6.8	0.01	4.2 × 10^-4^	NS
				48	35.0 ± 7.0	NS#	4.3 × 10^-5^	NS
				72	23.1 ± 4.6	3.6 × 10^-3^	4.3 × 10^-7^	3.6 × 10^-4^
CF1514		*muEx211* (intestine)		24	90.0 ± 2.0	1.7 × 10^-8^	NS	1.3 × 10^-7^
				48	38.3 ± 6.4	0.01	3.7 × 10^-5^	NS
				72	19.4 ± 4.5	0.01	1.5 × 10^-10^	3.0 × 10^-3^
CF1515		*muEx212* (muscle)		24	68.9 ± 2.9	4.5 × 10^-6^	1.4 × 10^-5^	1.4 × 10^-4^
				48	13.3 ± 3.3	NS#	6.6 × 10^-10^	NS
				72	5.6 ± 2.3	NS	2.3 × 10^-11^	NS

a NS = not significant (*p* > 0.01) as determined by one-way ANOVA with post hoc testing by the Bonferroni–Holm method. *p*-Values were for strain comparisons at the same time (h).

b Non-transgene = the strain with the same genetic background as the strain that it is being compared to, but this strain differs because it does not express a transgene; e.g. TJ356 animals were compared to N2 since both strain have a *WT* genetic background, but TJ356 contains a transgene while N2 does not.

c The *daf-16;daf-2* animals used here were obtained by selecting non-roller adult animals from those strains expressing the *daf-16*-tissue specific extra-chromosomal arrays (CF1442, CF1514, CF1515). Animals that did not roll were no longer expressing the extra-chromosomal array and were used as a control for determining the effects of the double mutation on the Se(IV)-induced motility deficits.

# *p* = 0.01when compared to the roller control strain (*daf-16;daf-2;yzIs71*; Fig. 3A, “#”).
