# Supplementary material for: The neurodegenerative effects of selenium are inhibited by FOXO and PINK1/PTEN regulation of insulin/insulin-like growth factor signaling in Caenorhabditis elegans
Source: Neurotoxicology. 2014 Mar;41(100):28–43. doi: 10.1016/j.neuro.2013.12.012 (PMC3979119; doi:10.1016/j.neuro.2013.12.012)
Supplement: Supplementary file 1 [file mmc2.zip › Transparency Document.pdf]

ELSEVIER

NeuroToxicology

Conflict of Interest Policy

The neurodegenerative effects of selenium are inhibited by FOXO and PINK1/PTEN regulation of insulin/insulin-like growth factor signaling in *Caenorhabditis elegans*

Annette O. Estevez, PhD

Declarations

NeuroToxicology requires that all authors sign a declaration of conflicting interests. If you have nothing to declare in any of these categories then this should be stated.

Conflict of Interest

A conflicting interest exists when professional judgement concerning a primary interest (such as patient's welfare or the validity of research) may be influenced by a secondary interest (such as financial gain or personal rivalry). It may arise for the authors when they have financial interest that may influence their interpretation of their results or those of others. Examples of potential conflicts of interest include employment, consultancies, stock ownership, honoraria, paid expert testimony, patent applications/registrations, and grants or other funding.

Please state any competing interests

I have nothing to declare.

Funding Source

All sources of funding should also be acknowledged and you should declare any involvement of study sponsors in the study design; collection, analysis and interpretation of data; the writing of the manuscript; the decision to submit the manuscript for publication. If the study sponsors had no such involvement, this should be stated.

Please state any sources of funding for your research

National Institutes of Environmental Health Sciences R21-ES012305

Signature (a scanned signature is acceptable, but each author must sign)

Print name

Annette Estevez, Ph.D.

ELSEVIER

NeuroToxicology

Conflict of Interest Policy

The neurodegenerative effects of selenium are inhibited by FOXO and PINK1/PTEN regulation of insulin/insulin-like growth factor signaling in *Caenorhabditis elegans*

Kathleen L. Morgan, PhD

Declarations

NeuroToxicology requires that all authors sign a declaration of conflicting interests. If you have nothing to declare in any of these categories then this should be stated.

Conflict of Interest

A conflicting interest exists when professional judgement concerning a primary interest (such as patient's welfare or the validity of research) may be influenced by a secondary interest (such as financial gain or personal rivalry). It may arise for the authors when they have financial interest that may influence their interpretation of their results or those of others. Examples of potential conflicts of interest include employment, consultancies, stock ownership, honoraria, paid expert testimony, patent applications/registrations, and grants or other funding.

Please state any competing interests

None

Funding Source

All sources of funding should also be acknowledged and you should declare any involvement of study sponsors in the study design; collection, analysis and interpretation of data; the writing of the manuscript; the decision to submit the manuscript for publication. If the study sponsors had no such involvement, this should be stated.

Please state any sources of funding for your research

Signature (a scanned signature is acceptable, but each author must sign)

Kathleen L Morgan

Print name

Kathleen L. Morgan, Ph.D.

ELSEVIER

NeuroToxicology

Conflict of Interest Policy

The neurodegenerative effects of selenium are inhibited by FOXO and PINK1/PTEN regulation of insulin/insulin-like growth factor signaling in *Caenorhabditis elegans*

Nathaniel J Szewczvk. Ph.D.

Declarations

NeuroToxicology requires that all authors sign a declaration of conflicting interests. If you have nothing to declare in any of these categories then this should be stated.

Conflict of Interest

A conflicting interest exists when professional judgement concerning a primary interest (such as patient's welfare or the validity of research) may be influenced by a secondary interest (such as financial gain or personal rivalry). It may arise for the authors when they have financial interest that may influence their interpretation of their results or those of others. Examples of potential conflicts of interest include employment, consultancies, stock ownership, honoraria, paid expert testimony, patent applications/registrations, and grants or other funding.

Please state any competing interests

Funding Source

All sources of funding should also be acknowledged and you should declare any involvement of study sponsors in the study design; collection, analysis and interpretation of data; the writing of the manuscript; the decision to submit the manuscript for publication. If the study sponsors had no such involvement, this should be stated.

Please state any sources of funding for your research

NIH, MRC, ARUK

Signature (a scanned signature is acceptable, but each author must sign)

Print name

Nathaniel J Szewczyk, Ph.D..

ELSEVIER

NeuroToxicology

Conflict of Interest Policy

The neurodegenerative effects of selenium are inhibited by FOXO and PINK1/PTEN regulation of insulin/insulin-like growth factor signaling in *Caenorhabditis elegans*

David Gems. Ph.D.

Declarations

NeuroToxicology requires that all authors sign a declaration of conflicting interests. If you have nothing to declare in any of these categories then this should be stated.

Conflict of Interest

A conflicting interest exists when professional judgement concerning a primary interest (such as patient's welfare or the validity of research) may be influenced by a secondary interest (such as financial gain or personal rivalry). It may arise for the authors when they have financial interest that may influence their interpretation of their results or those of others. Examples of potential conflicts of interest include employment, consultancies, stock ownership, honoraria, paid expert testimony, patent applications/registrations, and grants or other funding.

Please state any competing interests

I have nothing to declare.

Funding Source

All sources of funding should also be acknowledged and you should declare any involvement of study sponsors in the study design; collection, analysis and interpretation of data; the writing of the manuscript; the decision to submit the manuscript for publication. If the study sponsors had no such involvement, this should be stated.

Please state any sources of funding for your research

Wellcome Trust, European Union

Signature (a scanned signature is acceptable, but each author must sign)

Print name

David Gems, Ph.D.

ELSEVIER

NeuroToxicology

Conflict of Interest Policy

The neurodegenerative effects of selenium are inhibited by FOXO and PINK1/PTEN regulation of insulin/insulin-like growth factor signaling in *Caenorhabditis elegans*

Miguel Estevez, M.D., Ph.D.

Declarations

NeuroToxicology requires that all authors sign a declaration of conflicting interests. If you have nothing to declare in any of these categories then this should be stated.

Conflict of Interest
A conflicting interest exists when professional judgement concerning a primary interest (such as patient's welfare or the validity of research) may be influenced by a secondary interest (such as financial gain or personal rivalry). It may arise for the authors when they have financial interest that may influence their interpretation of their results or those of others. Examples of potential conflicts of interest include employment, consultancies, stock ownership, honoraria, paid expert testimony, patent applications/registrations, and grants or other funding.

Please state any competing interests

I have nothing to declare.

Funding Source
All sources of funding should also be acknowledged and you should declare any involvement of study sponsors in the study design; collection, analysis and interpretation of data; the writing of the manuscript; the decision to submit the manuscript for publication. If the study sponsors had no such involvement, this should be stated.

Please state any sources of funding for your research

Jim Himelic Foundation; Southern Arizona Foundation; Keatings Institute; National Institutes of Environmental Health Sciences R21-ES012305; Career Development Award through the Office of Research and Development, Department of Veterans Affairs

Signature (a scanned signature is acceptable, but each author must sign)

Print name

M Estevez

Miguel Estevez, M.D., Ph.D.
